# COVID-19 Deaths in the United States: Shifts in Hot Spots over the Three Phases of the Pandemic and the Spatiotemporally Varying Impact of Pandemic Vulnerability

**DOI:** 10.3390/ijerph18178987

**Published:** 2021-08-26

**Authors:** Yoo Min Park, Gregory D. Kearney, Bennett Wall, Katherine Jones, Robert J. Howard, Ray H. Hylock

**Affiliations:** 1 Department of Geography, Planning and Environment, East Carolina University, Greenville, NC 27858, USA; howardro@ecu.edu; 2 Department of Public Health, Brody School of Medicine, East Carolina University, Greenville, NC 27834, USA; kearneyg@ecu.edu (G.D.K.); joneska@ecu.edu (K.J.); 3 Vidant Medical Center, Greenville, NC 27835, USA; Bennett.Wall@vidanthealth.com; 4 Department of Health Services and Information Management, East Carolina University, Greenville, NC 27834, USA; hylockr@ecu.edu

**Keywords:** coronavirus, COVID-19 mortality, social vulnerability, space-time cube, emerging hot spot analysis, geographically and temporally weighted regression, pandemic vulnerability index, health disparity

## Abstract

The geographic areas most impacted by COVID-19 may not remain static because public health measures/behaviors change dynamically, and the impacts of pandemic vulnerability also may vary geographically and temporally. The nature of the pandemic makes spatiotemporal methods essential to understanding the distribution of COVID-19 deaths and developing interventions. This study examines the spatiotemporal trends in COVID-19 death rates in the United States from March 2020 to May 2021 by performing an emerging hot spot analysis (EHSA). It then investigates the effects of the COVID-19 time-dependent and basic social vulnerability factors on COVID-19 death rates using geographically and temporally weighted regression (GTWR). The EHSA results demonstrate that over the three phases of the pandemic (first wave, second wave, and post-vaccine deployment), hot spots have shifted from densely populated cities and the states with a high percentage of socially vulnerable individuals to the states with relatively relaxed social distancing requirements, and then to the states with low vaccination rates. The GTWR results suggest that local infection and testing rates, social distancing interventions, and other social, environmental, and health risk factors show significant associations with COVID-19 death rates, but these associations vary over time and space. These findings can inform public health planning.

## 1. Introduction

The emergence of the coronavirus disease 2019 (COVID-19) has caused unprecedented challenges to global public health and healthcare systems since its onset in late 2019. Several studies have reported that the pandemic has disproportionately impacted socially vulnerable populations, including the elderly, racial/ethnic minorities, low-income communities, people with low educational attainment, the uninsured population, people with chronic comorbidities and underlying health conditions, and other underserved communities [1,2,3,4]. Protecting these groups is paramount due to their increased risk of COVID-19 mortality [5,6]. Delivering effective interventions that address health disparities related to COVID-19 requires identifying vulnerable communities that lack the resources necessary to respond to the pandemic and understanding the social determinants of health and structured inequalities that these communities face.

Extensive medical and epidemiological research has been conducted to uncover the characteristics of the virus and the impact of social vulnerability on COVID-19 incidence/mortality as well as to find a cure using various mathematical or statistical models [7]. However, efforts to understand the dynamics of the pandemic in the spatiotemporal dimension using spatially and temporally explicit methods have been relatively limited. Yet, similar to most other infectious diseases, the COVID-19 pandemic intrinsically has spatial and temporal characteristics because the dynamic interactions between the virus, human hosts, and the environmental factors that facilitate transmission shape the spatiotemporal variations in the disease risks [8].

Geographically close communities tend to exhibit similar COVID-19 mortality rates as they may share common local risk factors (i.e., similar social and physical environments) and the disease transmission typically occurs through close contact [9]. To characterize the spatial nature of the COVID-19 pandemic, several studies have utilized spatial statistical methods, such as the Getis-Ord Gi* statistic [10] or local Moran’s *I* [11] to identify the locations of spatial clustering of COVID-19 cases/deaths (e.g., [7,12,13]). Others have used geographically weighted regression (GWR) [14] or modified versions of GWR to explore local variations in the associations between COVID-19 cases/deaths and potential influencing factors (e.g., [15,16,17]). Although these spatial methods can account for the spatial structure of data, the investigated processes are assumed to be constant over time in these methods. However, the geographic areas most affected by COVID-19 may not remain static as some risk factors dynamically change over time [6,18,19]. This indicates that the relationships between COVID-19 mortality and vulnerability factors may vary not only spatially but also temporally. The spatiotemporal nature of the COVID-19 pandemic and changing public health measures and behaviors make spatially and temporally explicit methods critical for understanding the behavior of the disease and effective public health planning.

This study identifies the spatiotemporal trends in COVID-19 mortality in the United States using space-time pattern mining techniques. It then investigates the spatiotemporally varying impacts of pandemic vulnerability factors on COVID-19 mortality using a geographically and temporally weighted regression (GTWR) model to capture both spatial and temporal non-stationarity in the data. This is the first study using spatiotemporally explicit methods to examine the association between COVID-19 deaths and the individual data components of the COVID-19 Pandemic Vulnerability Index (PVI) [20], which was recently developed by researchers from the National Institute of Environmental Health Sciences (NIEHS), North Carolina State University, and Texas A&M University. Many previous studies have focused on traditional census variables integrated into the Social Vulnerability Index (SVI) of the U.S. Centers for Disease Control and Prevention (CDC), such as socioeconomic characteristics, household composition, minority status, housing types, and access to transportation. However, the static characteristics of these variables pose a significant challenge to determining vulnerability associated with COVID-19 using these variables because they do not sufficiently represent the highly dynamic nature of the pandemic. The PVI was developed in recognition of the need for a more comprehensive measure that integrates not only the traditional social vulnerability factors but also the pandemic-related time-dependent factors, such as current infection rates, COVID-19 testing rates, social distancing scores, and the rate of disease spread [20]. It also integrates health-related factors that have been reported to be associated with more severe outcomes from COVID-19 infection, such as the prevalence of comorbidities and healthcare resource allocation.

## 2. Materials and Methods

### 2.1. Data

The daily COVID-19 death counts and county-level PVI data for the contiguous United States were obtained from the PVI’s GitHub repository—specifically, the PVI Model 11.2.1. This study uses data from 1 March 2020 to 31 May 2021 (15 months). COVID-19 death rates (deaths per 100,000 people) are calculated using county-level population estimates from the U.S. Census Bureau American Community Survey (ACS) 2014–2018 (5-year) data. The PVI comprises two dynamic domains directly related to COVID-19—(1) infection rate and (2) intervention measures—and two static domains related to general social vulnerability for public health and natural/human-caused disasters—(1) population concentration and (2) health and environment. The dynamic variables are updated daily. Many social vulnerability factors used in the PVI are derived from the CDC SVI.

Table 1 presents a description of the individual variables included in each domain. These variables can help account for differential place-based risk factors associated with COVID-19, such as racial disparities, socioeconomic disparities, unequal allocation of health resources, health disparities, and comorbidities known to increase COVID-19 mortality rates [20]. Further details about the PVI variables, rationale for their inclusion, and data sources are available in Marvel et al. [20] and NIEHS [21].

Because GTWR is computationally demanding and produces several mappable results for a set of explanatory variables for each time step, the feasibility of computation and the effective presentation of analysis results are the major challenges of GWR/GTWR applications [22,23]. Therefore, the data are aggregated into three timesteps (Period 1: March–July 2020; Period 2: August–December 2020; Period 3: January–May 2021) for ease of computation and presentations. Period 1 represents an early phase of the pandemic. Period 2 represents a second wave. Period 3 represents a period after the impact of COVID-19 vaccination becomes effective.

### 2.2. Emerging Hot Spot Analysis (Space-Time Pattern Mining)

An emerging hot spot analysis (EHSA) is performed on COVID-19 death rates to identify their spatiotemporal clusters at a county level. In general, a hot spot indicates an area with a number or intensity of events that is higher than average. This study defines a COVID-19 hot spot as an area that exhibits a concentration of high death rates. Likewise, a COVID-19 cold spot indicates a spatial cluster of low death rates. In a COVID-19 hot spot, the pandemic has a more serious impact on the population than in other areas. One of the most common methods for detecting spatial clusters is the Getis-Ord Gi* statistic [10]. The statistic investigates where high/low values cluster spatially and whether the observed clusters are statistically significant. A county is classified as a hot spot when (1) it has a high value and its neighboring counties also have similarly high values and (2) this clustering pattern is unlikely to be the result of random chance. The equation for the Getis-Ord Gi* statistic is as follow:

(1) $$G_{i}^{*}=\frac{\sum_{j=1}^{n}w_{i,j}x_{j}-\bar{X}\sum_{j=1}^{n}w_{i,j}}{S\sqrt{\frac{\left[n\sum_{j=1}^{n}w_{i,j}^{2}-{\left(\sum_{j=1}^{n}w_{i,j}\right)}^{2}\right]}{n-1}}}$$

where ***i*** is a location at which the $G_{i}^{*}$ is being calculated, $x_{j}$ is the attribute value for a location ***j***, and $w_{i,j}$ denotes the spatial weight between the locations ***i*** and ***j***. In this study, the *K*-nearest neighbors method with 10 for the number of closest neighbors to include in the analysis for the location ***i*** is used to define the spatial weight matrix. ***n*** is the number of locations in the study area, and:

(2) $$\bar{X}=\frac{\sum_{j=1}^{n}x_{j}}{n}$$

(3) $$S=\sqrt{\frac{\sum_{j=1}^{n}x_{j}^{2}}{n}-{\left(\bar{X}\right)}^{2}}$$

**Figure 1:**
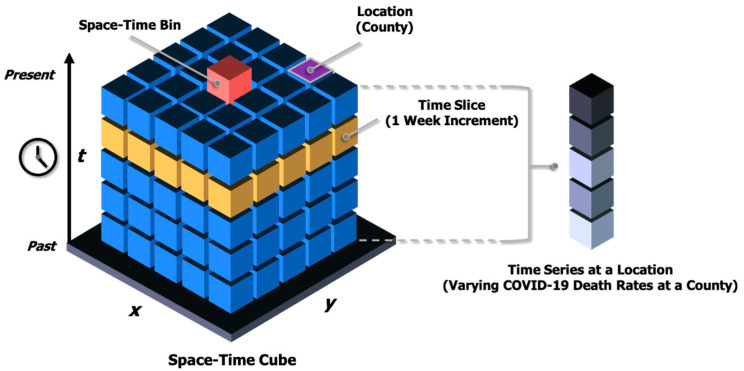
The structure of a space-time cube.

The EHSA extends the Getis-Ord Gi* statistic to incorporate the temporal dimension of data [24]. It evaluates not only the location/degree of the spatial clustering of an event but also the temporal trends across the time series by combining two statistical methods, the Getis-Ord Gi* statistic and the Mann-Kendall trend test. The input data set to EHSA are space-time cubes in a netCDF data structure (i.e., a data format for storing multidimensional scientific data). A space-time cube is a three-dimensional (3-D) cube that consists of space-time bins (*x*, *y*, *t*) in which the *x* and *y* dimensions represent space and the *t* dimension represents time (Figure 1). Data are aggregated into space-time bins, where observations within the same spatial range and time period fall into the same bin. This study defines a county as a spatial range and one week as a time period for each bin. Each bin has a unique location ID (i.e., geographic identifier of a county) and holds aggregated values, such as the number of points or other summary statistics of specified attributes (e.g., sum, mean, maximum, minimum, standard deviation, and median).

Taking the space-time cube as input, the Getis-Ord Gi* statistic is calculated to assign each bin a hot spot bin classification with an associated *z*-score and *p*-value. A bin is classified as a hot spot if its local summary statistic is significantly higher than the expected local statistic (i.e., the local statistic calculated based on the assumption of complete spatial randomness) and if that difference is too large to be the result of a random process. The observed local statistic for the bin is calculated using the values of its spatiotemporal neighbors. Once all bins are assigned a hot spot bin classification, the Mann–Kendall trend test is used to evaluate hot/cold spot trends at each location (i.e., county) and classify counties into 17 categories, including new, consecutive, intensifying, persistent, diminishing, sporadic, oscillating, historical hot/cold spot, and no pattern detected. Table 2 presents the definitions of hot/cold spot trend classifications. The outputs are visualized in two-dimensional (2-D) and 3-D maps to present the changes of the COVID-19 landscape over time.

### 2.3. Geographically and Temporally Weighted Regression

While the relationships between a dependent variable and explanatory variables are constant in global regression models, such as ordinary least squares (OLS), GWR, a local form of linear regression, allows those relationships to vary over space to account for spatial non-stationarity in parameters. GTWR extends GWR to incorporate a time dimension to capture spatial and temporal heterogeneity simultaneously [25]. In GTWR, it is assumed that observations that are geographically and temporally close to the observation *i* have a larger influence on the parameter estimation for the observation *i* than the distant observations [25]. Therefore, GTWR requires defining spatial and temporal “closeness” among observations in a space-time coordinate system by constructing a spatiotemporal weight matrix. A regression model is calibrated by assigning greater weights to data points closer to the observation *i* in a space-time coordinate system while estimating parameters for the observation *i*. By producing location- and time-specific parameters, GTWR explains geographically and temporally varying relationships. Therefore, unlike global models in which one equation is applied to all observations, a separate equation is derived for each observation in GTWR to capture local effects. A typical GTWR model can be expressed as follows:

(4) $$Y_{i}=\beta_{0}\left(u_{i},v_{i},t_{i}\right)+\sum_{k}^{p-1}\beta_{k}\left(u_{i},v_{i},t_{i}\right)X_{i,k}+\varepsilon_{i}$$

where $Y_{i}$ is the dependent variable at a space-time location ***i***, $X_{i,k}$ is a set of explanatory variables (***k*** = 1, 2, …, *p*−1) at the location ***i***, ***p*** is the total number of parameters to be estimated. $\left(u_{i},v_{i},t_{i}\right)$ denotes the geographic coordinates of the location ***i*** $\left(u_{i},v_{i}\right)$ at a given time $\left(t_{i}\right)$. $\beta_{0}\left(u_{i},v_{i},t_{i}\right)$ and $\beta_{k}\left(u_{i},v_{i},t_{i}\right)$ represent the intercept and the coefficient for the explanatory variable *k* at the space-time location ***i***, respectively. $\varepsilon_{i}$ is an error term for the location ***i***.

Because the units of spatial and temporal distance are different, scale factors ***λ*** and ***μ*** are applied to prevent either spatial distance $d_{space}$ or temporal distance $d_{time}$ from having a dominant influence on the calculation of spatiotemporal distance $d_{space–time}$ (Equation (5)). We optimized the scale factors using cross-validation to improve the goodness-of-fit (*R^2^*). In this study, ***λ*** of 1 and ***μ*** of 0.5418 are used. More details of the technical implementation of GTWR can be found in Huang et al. [25].

(5) $$d_{space–time}=\lambda d_{space}+\mu d_{time}$$

All explanatory variables included in GTWR should be statistically significant [25]. Prior to the GTWR analysis, a global OLS model is thus formed to assess the significance of each variable. The dependent variable is the COVID-19 death rate; these rates are log-transformed to satisfy the parametric requirement for normality. The explanatory variables represent the best fitting (in terms of model performance) subset of the 18 PVI variables using a bi-directional, stepwise algorithm (i.e., forward and backward selection). All data analyses and mapping were performed using ArcGIS Pro (version 2.7.1) and R statistical software (version 4.0.3).

## 3. Results

### 3.1. Spatiotemporal Trend in COVID-19 Mortality Rates

**Figure 2:**
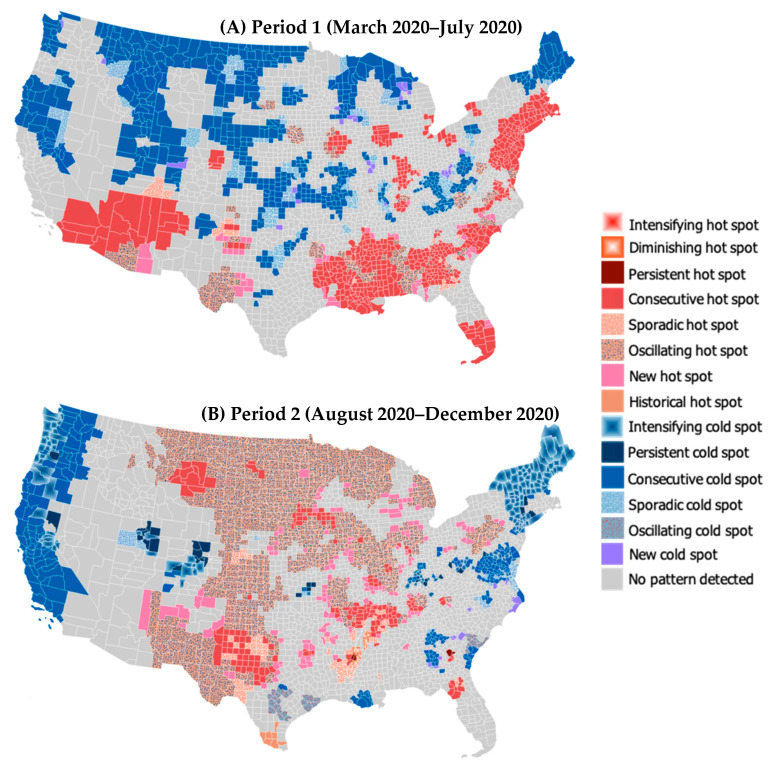

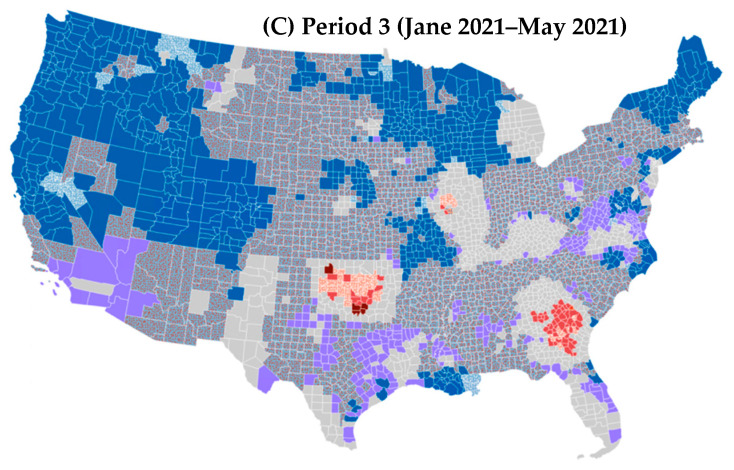
2-D geovisualization of hot and cold spot trends of COVID-19 mortality rates in Period 1 (**A**), Period 2 (**B**), and Period 3 (**C**).

A space-time cube that includes a total of 68,376 bins (3108 locations × 22 time steps) is generated for each period and used as input in the EHSA. The results of the EHSA suggest that the hot/cold spot patterns of COVID-19 death rates are substantially different between the three periods (Figure 2). In the early phase of the pandemic (Figure 2A), most hot spots of COVID-19 death rates were found in the Deep South (i.e., Louisiana, Mississippi, Alabama, Georgia, and South Carolina), South Florida, and the Southwestern states. The high death rates in these regions may be due to the high percentage of socially vulnerable populations, such as uninsured residents, people living in poverty, individuals with jobs that require working in close contact, and elderly people, as well as the lack of binational public health strategies between the United States and Mexico and limited public testing. High death-rate clusters are also observed in the large urban communities in the Northeast megalopolis (Boston–New York–Washington DC corridor) due to high population density (making social distancing difficult), the high number of confirmed cases, super-spreading events early in the pandemic, and high traffic and travel levels.

During the second wave of the pandemic (Figure 2B), however, many of these initial hot spots became not statistically significant (no pattern detected) or cold spots. This change may have occurred because these regions had a higher percentage of people with antibodies from previous infection, stronger quarantine requirements were implemented for travelers than other regions, and many people left the cities for suburbs, smaller metropolitan areas, and rural communities. During this period, communities in the Great Plains (from Montana, North Dakota to West Texas) and Midwest (e.g., Minnesota, Wisconsin, Michigan, Iowa, Illinois, Indiana, and Missouri) were more severely affected. Overall, the number of hot spots increased from 767 to 1147. Many new, oscillating, sporadic, and consecutive hot spots were detected across the country due to the relative relaxing of quarantine guidelines and social distancing interventions, the reopening of businesses/schools, and cold weather.

Interestingly, many of the oscillating hot spots in Period 2 became oscillating cold spots in Period 3 (Figure 2C), indicating the recent change from high- to low-value clustering. These changes demonstrate the substantial decline in COVID-19 death rates across the country and highlight the effectiveness of vaccination in protecting people against the infection and adverse outcomes from COVID-19. However, even when 74% of the counties became cold spots during this period, persistent, intensifying, consecutive, and sporadic hot spots of COVID-19 death rates were identified in certain counties in Georgia, Oklahoma, and Illinois. The percentages of the population fully vaccinated in these states are below the national average (45.3%) as of June 23, 2021, according to the CDC (Georgia: 35.4%; Oklahoma: 37.4%; and Illinois: 44.5%). It is worth noting that Richmond County (Georgia)—in which the majority of the population is Black or African American (55.7%) and more than one-third of the population faces limited healthcare access and poverty—is the sole intensifying hot spot in the country. This suggests that without appropriate public health interventions, the county may experience a surge in COVID-19 deaths in the future.

**Figure 3:**
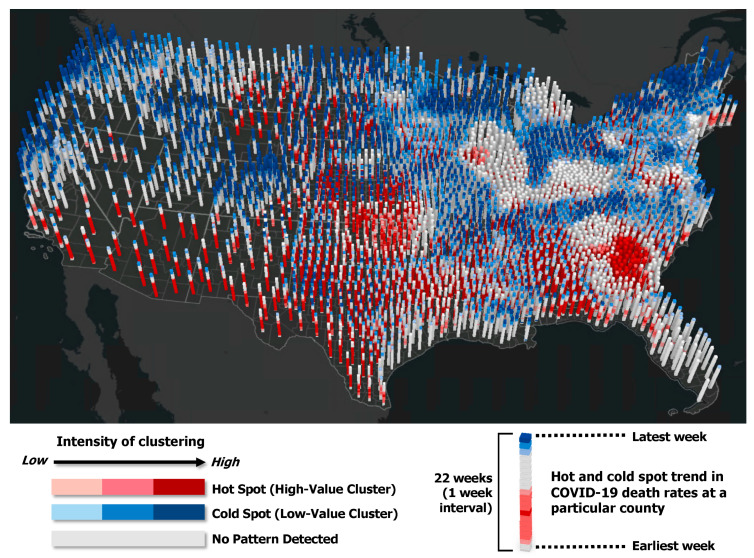
3-D geovisualization of hot and cold spot trends of COVID-19 mortality rates in Period 3.

Figure 3 illustrates the weekly hot/cold spot trend during Period 3 in 3-D, where each column of stacked bins represents a time series of hot/cold spot patterns of COVID-19 death rates at the county level, with the latest week on the top. The darker red or blue colors indicate more intense clustering of high or low death rates. The 3-D map shows that most previous hot spots became cold spot or not significant over time, with a few exceptions (counties in Georgia, Oklahoma, and Illinois).

### 3.2. Results of the Global Regression Model

Fifteen explanatory variables are chosen as a result of the stepwise selection. These variables do not have a multicollinearity issue (variance inflation factor [VIF] values ranging from 1.07 to 2.90). Table 3 shows the parameter estimates of the global OLS model. All model coefficients are statistically significant at a 5% significance level, except the percentage of Native Americans at the margin of statistical significance (*p* = 0.057). The adjusted *R^2^* of the OLS model is 0.54 (*p* < 0.001).

The OLS analysis results suggest that COVID-19 mortality rates are positively associated with infection prevalence, social distancing scores (with a higher score indicating more travel and contact, i.e., less social distancing), the COVID-19 testing metric (with a higher value indicating less testing), traffic volume, residential density, air pollution, premature death, and the percentages of Black, Native Americans, uninsured, diabetic, and aged 65 and above populations. The rate of disease spread, percentage of current smokers, and summation of hospital beds are negatively associated with mortality rates. The inverse relationship with the disease spread metric may be due to spatial and temporal non-stationarity in the data or unreliability in the metric. The metric is calculated based on the assumption that COVID-19 has an incubation period of 14 days, but the true length of the incubation period has not been fully established; thus, further research is needed to validate the metric’s reliability. The result for smoking may also be impacted by spatial and temporal non-stationarity but should be interpreted with caution due to mixed findings in the literature [26]. While several studies have suggested a positive association between smoking and adverse health outcomes from COVID-19 (e.g., [27,28,29]), other reports have indicated an inverse relationship or no relationship (e.g., [30,31]). Therefore, more extensive, carefully designed patient-level studies with sufficient sample sizes in various geographical settings are required to establish the effect of smoking on the increased severity of the disease or mortality [26].

### 3.3. Results of the GTWR Model

**Figure 4:**
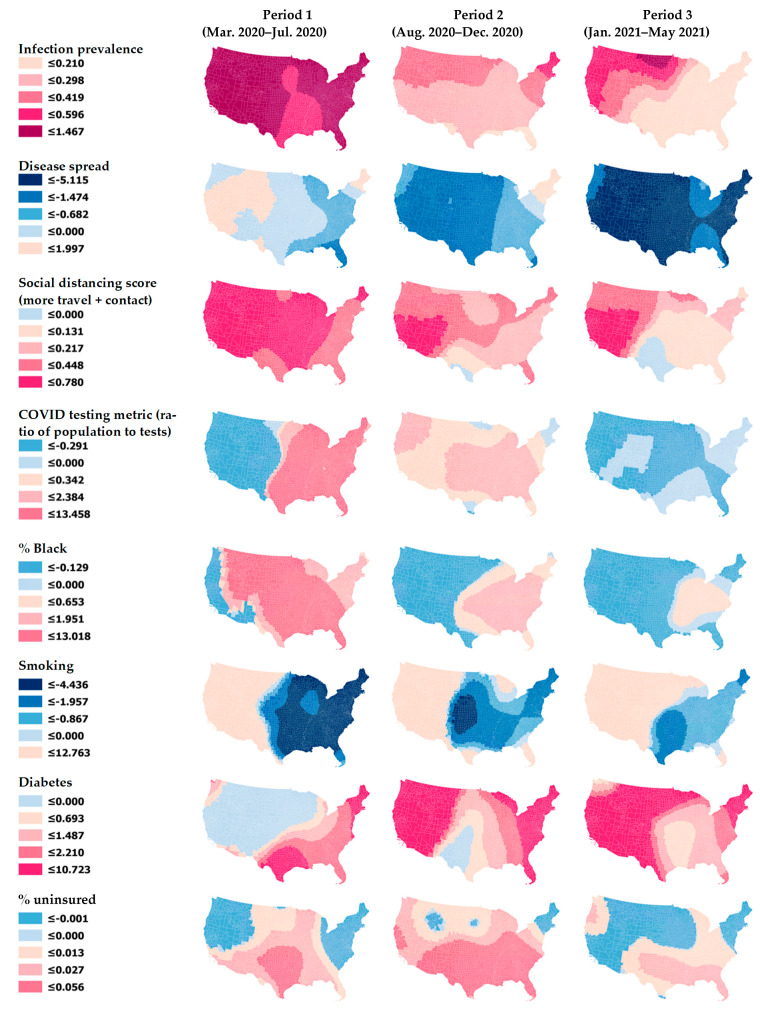
Spatially and temporally varying relationships between COVID-19 mortality rates and selected PVI variables.

The GTWR model has a higher adjusted *R^2^* of 0.68 compared to the 0.54 of the global model. This indicates that the GTWR has improved the model performance by explaining an additional 14% of the total variance of COVID-19 death rates by accounting for the spatial and temporal structure of the data. The reduction in the corrected Akaike Information Criterion (AICc) value (from 27,964 for the OLS model to 24,601 for the GTWR model) also demonstrates the superiority of the GTWR model over the OLS model. The GTWR returns separate, mappable coefficients for all explanatory variables for each time period, but Figure 4 includes maps of only eight selected variables due to space limitations. The six statistics for the varying coefficients of all the variables are presented in Table 4.

Figure 4 and Table 4 show how the coefficients of each variable fluctuate over space and time. Taking the social distancing variable as an example, the effect of less social distancing (i.e., a higher social distancing score reflects more travel and contact) on COVID-19 mortality rates was strongest in the early phase of the pandemic, but it was later attenuated as the pandemic wore on and the compliance waned and became uneven (Figure 4). For COVID-19 testing, the lower quartile of the coefficient is −0.291, while the upper quartile is 2.384 (Table 4). Although its global coefficient (0.301) indicates that less testing is associated with higher mortality rates (note that the testing metric used in this study is the ratio of population to tests, so a higher value means fewer people are getting tested), some local coefficients that are negative suggest that more testing is associated with high mortality rates at certain times (Period 3) and in particular regions (Western United States in Period 1) (Figure 4). Similarly, while the coefficient sign of smoking rates is negative in the global model, the GTWR model suggests that smoking has a harmful effect on COVID-19 deaths in the Western United States. This positive relationship is present in all three periods (Figure 4). The impact of being uninsured is also not constant across space due to different social and environmental characteristics across the country, placing communities in the Southern United States at a higher risk of adverse outcomes than other regions in the United States from the infection in all three periods.

## 4. Discussion and Conclusions

This study investigates the spatiotemporal trends and geographic disparities in COVID-19 mortality from 1 March 2020 to 31 May 2021 by identifying emerging hot and cold spots of death rates over the three phases of the pandemic. The overall hot spot patterns found in Periods 1 and 2 are similar to those in a study that examined the monthly trend of COVID-19 deaths using data up to December 2020 [32]. This study also suggests spatiotemporally varying relationships between COVID-19 mortality and the pandemic-related social vulnerability factors by incorporating the spatial and temporal dimensions into regression analysis. The findings of the GTWR analysis are generally consistent with the study by Neelon et al. [6] examining data up to December 2020, which found that COVID-19 deaths were positively associated with the percentage of individuals in poor or fair health with comorbidities as in a prior study [33], and with the average air quality measured by PM_2.5_ concentrations as in the paper of Wu et al. [34]. Neelon et al. also found that a higher percentage of adult smokers was associated with a lower rate of COVID-19 deaths [6], which is supportive of our finding. However, while they found a positive relationship between testing rates and COVID-19 death rates, our OLS regression result suggests the inverse relationship. This discrepancy may be because the effect varies over space and time as indicated in our GTWR analysis results. A recent study [32] supports our conclusion on the spatial and temporal non-stationarity of COVID-19 testing. The findings demonstrate a dynamic impact of the COVID-19 pandemic on vulnerable communities. As the pandemic is likely to continue, it is critical to consider the spatiotemporal trends in COVID-19 death rates as well as the distribution of emerging and persistent risk factors to identify the communities in greatest need in a timely manner and to make public health decisions for resource allocations.

Several limitations of this study should be taken into consideration. First, this study obtains the COVID-19 death counts from the USA Facts website (which is linked to the PVI database). However, the death counts are likely to be undercounted due to the lack of consensus on which mortalities should be considered deaths from COVID-19 and the complexity of determining the cause of death in general [35]. The inaccuracy in determining the county of residence for deaths, under-testing, and high proportions of asymptomatic cases [36,37,38] may also have influenced the mortality data. Therefore, the reporting bias in the data and regional variations in reporting rates may have influenced the analysis results. Second, similar to most spatial analyses, this study is subject to the modifiable areal unit problem (MAUP), which occurs when an arbitrarily delineated spatial unit is used to analyze a geographically continuous phenomenon [39]. Because counties are artificially defined, different associations may be found if different spatial units are used. However, the analysis in this study is restricted to the county level because the PVI variables are not available at a smaller spatial unit. Future studies might consider using the Vaccine Priority Index (VPI) [40] to examine the association between COVID-related social vulnerability and cases/deaths at a census-tract level and identify “high-risk, high priority” census tracts. Nonetheless, counties are useful administrative units for guiding policy planning and resource allocation. Similarly, the results may be influenced by how the time frame is defined. This issue, known as the modifiable temporal unit problem (MTUP) [41], would be an interesting future research topic. Future studies might replicate our work to investigate the effects of both MAUP and MTUP.

A final limitation is that this study assumes that people are only influenced by the social and environmental characteristics of the county in which they reside and that residents in the same county face the same risk of COVID-19 mortality. However, many people may have traveled across the counties to work, shop, engage in leisure activities, and visit family and friends, although mobility was relatively limited during the pandemic due to the travel restrictions. It is worth noting that ignoring human mobility in studies on contextual determinants of health may produce misleading findings due to uncertainty regarding the spatial and temporal contexts in which a person experiences contextual influence [42]. Relatedly, the effect of the PVI variables (neighborhood variables) on COVID-19 mortality may decrease if individual mobility is considered due to the neighborhood effect averaging problem (NEAP) [43]. Individual-level, daily mobility pattern data for COVID-19 patients can be used to address these methodological problems. However, such fine-scale location data are not available in the United States due to geoprivacy concerns [44].

Despite the limitations above, this study contributes to the literature by considering the spatiotemporal characteristics of the ongoing pandemic to reveal the structured disparities in COVID-19 mortality in the United States. It also offers public health authorities the increased knowledge of spatial and temporal trends of COVID-19 deaths, which is crucial to the development of targeted prevention and intervention strategies of the ongoing pandemic. As new viral strains are detected and cross geographical boundaries, continued surveillance and data-driven decisions will remain vital elements to inform policy decisions and communicate risk to protect communities. Based on the GTWR regression results, social distancing was effective across all regions in Period 1 in reducing mortality. However, the effectiveness of the intervention seemed to weaken and vary regionally as the pandemic wore on, possibly because community spread was already too broad for distancing to matter, or because the increased vaccination rates made the impact of social distancing less apparent. The infection prevalence, a measure of temporal case clusters, is high when there is a “bubble” of many cases over a two-week window. In Period 1, the infection prevalence was positively associated with death rates across all regions, indicating that when cases rates were high, death rates were also high. By Period 3, the relationship was weaker in the Eastern and Southern United States, but still strong in the upper Midwest. This shift may reflect regional or local differences in health system capacity and adaptation, as well as a better understanding of the disease and more treatment options. Some regions or systems may have developed the capacity to respond effectively to case “bubbles” and lower the mortality rates. Particularly in the context of the highly transmissible variants, it may be worthwhile to investigate local variation in the infection prevalence along with how localities were able to quickly administer vaccines and deploy additional prevention strategies to protect populations at high risks for severe illness or death (e.g., people aged 65 and over or people with underlying health conditions), as a way to look for successful health system adaptations.

Advanced geographical approaches will continue to play a critical role in identifying the locations of disease clustering and vulnerable populations and in implementing effective public health action. The findings of this study can inform future epidemiological studies and health-disparity research and contribute to public health decision-making to prevent disease and mitigate health disparity during the current pandemic and future crises.

## Figures and Tables

**Table 1:** A description of the PVI variables that comprise the four major domains [21].

Data Components in Four Major Domains	Characteristic	Description
**1. Infection Rate**		
Infection prevalence (Transmissible cases)	Dynamic	Cases from the last 14 days divided by population size (counts are obtained from the USA Facts website). The greater this metric, the more likely the virus is to continue to spread.
Rate of disease spread	Dynamic	New cases (for the last 14 days) divided by total active cases (counts are obtained from the USA Facts website). The value is close to 1 during exponential growth phase and declines linearly to 0 over the two-week incubation period if there are no new cases.
**2. Intervention Measures**		
Social distancing score	Dynamic	Changes in overall distance traveled and nonessential visits relative to previous year. A higher score indicates more travel and contact, i.e., less social distancing (derived from Unacast mobile phone data).
COVID-19 testing metric (state-level)	Dynamic	Population divided by the number of COVID-19 tests (the inverse of the total tests per population). A higher number indicates that fewer people are getting tested, i.e., lower testing rate (derived from the COVID Tracking Project [Atlantic Monthly Group 2020]).
**3. Population Concentration**		
Daytime population density	Static	The greater daytime population density, the more likely the disease will spread (derived from 2018 CDC SVI).
Baseline traffic	Static	Average traffic volume per meter of major roadways (derived from 2018 Environmental Justice Screening and Mapping Tool of the U.S. Environmental Protection Agency).
Residential density (SVI housing type and transportation score)	Static	One of the four themes in the SVI (derived from 2018 CDC SVI). Calculated based on families in multi-unit structures, mobile homes, overcrowding (persons > rooms), no vehicle, and persons in group quarters.
**4. Health and Environment**		
% Black	Static	Percentage of Black or African American.
% Native Americans	Static	Percentage of American Indian or Alaska Native.
Air pollution	Static	Average daily levels of particulate matter 2.5 (µg/m^3^) (derived from 2014 Environmental Public Health Tracking Network).
% aged 65 and over	Static	Percentage of population aged 65 and older (derived from 2014 to 2018 ACS).
Premature death	Static	Years of potential life lost before age 75 per 100,000 (derived from 2016 to 2018 National Center for Health Statistics: Mortality Files).
Smoking	Static	Percentage of adult smokers (derived from 2017 Behavioral Risk Factor Surveillance System).
Diabetes	Static	Percentage of adults with diabetes (aged 20 and order) (derived from 2016 U.S. Diabetes Surveillance System).
Obesity	Static	Percentage of population aged 20 and older with a body mass index ≥ 30 kg/m^2^.
% uninsured	Static	Percentage of population uninsured (derived from 2018 CDC SVI).
SVI socioeconomic status score	Static	One of the four themes in the SVI (derived from 2018 CDC SVI). Calculated based on percent below poverty, percent unemployed, income, and percent with no high school diploma.
Hospital beds	Static	Hospital beds in general medical and surgical hospitals (derived from the Homeland Infrastructure Foundation-Level Data).

**Table 2:** Definitions of hot and cold spot trend classifications [24].

Pattern	Definition
Intensifying hot (cold) spot	A location in which at least 90% of time steps present clustering of high (low) values and the intensity of clustering is *increasing* over time.
Diminishing hot (cold) spot	A location in which at least 90% of time steps present clustering of high (low) values and the intensity of clustering is *decreasing* over time.
Persistent hot (cold) spot	A location in which at least 90% of time steps present clustering of high (low) values *without* an increasing or decreasing trend in the intensity of clustering.
Consecutive hot (cold) spot	A location with a single *continuous* run of clustering of high (low) values for less than 90% of all time steps.
Sporadic hot (cold) spot	A location in which clustering of high (low) values is *on again and off again* over time *without* a history of clustering of low (high) values for all time steps.
Oscillating hot (cold) spot	A location with clustering of high (low) values for the latest time step *with* a history of clustering of low (high) values during previous time steps.
New hot (cold) spot	A location in which clustering of high (low) values has *never* been identified *except* for in the latest time step.
Historical hot (cold) spot	A location in which clustering of high (low) values has always been identified *except* for in the latest time step.
No pattern detected	A location with *no* statistically significant hot or cold spot pattern detected.

**Table 3:** Parameter estimates of the global OLS model.

Parameter	Coefficient Estimate	Standard Error	*t*-Statistic	*p*-Value
Intercept	0.395 **	0.146	2.703	0.007
Infection prevalence	0.305 ***	0.006	52.929	<0.001
Disease spread	−1.594 ***	0.102	−15.697	<0.001
Social distancing score	0.205 ***	0.023	8.850	<0.001
COVID-19 testing metric	0.301 ***	0.042	7.151	< 0.000
Baseline traffic	1.739 × 10^−4^ ***	4.274 × 10^−5^	4.069	<0.001
Residential density	0.106 *	0.049	2.167	0.030
% Black	1.299 ***	0.102	12.779	<0.001
% Native Americans	0.434 .	0.228	1.905	0.057
Air pollution	0.084 ***	0.008	9.987	<0.001
% aged 65 and over	0.019 ***	0.003	5.876	<0.001
Premature death	3.010 × 10^−5^ ***	7.577 × 10^−6^	3.973	<0.001
Smoking	−2.199 ***	0.526	−4.183	<0.001
Diabetes	1.499 ***	0.387	3.874	<0.001
% uninsured	0.028 ***	0.003	9.749	<0.001
Hospital beds	−11.720 ***	2.779	−4.219	<0.001

‘***’ *p* ≤ 0.001; ‘**’ *p* ≤ 0.01; ‘*’ *p* ≤ 0.05; ‘.’ *p* ≤ 0.1.

**Table 4:** Parameter estimates of the GTWR model.

Parameter	Mean	Minimum	Lower Quartile	Median	Upper Quartile	Maximum
Intercept	0.208	−2.793	−0.861	0.126	1.534	3.962
Infection prevalence	0.419	0.064	0.210	0.298	0.596	1.466
Disease spread	−3.504	−27.958	−5.115	−1.474	−0.682	1.997
Social distancing score	0.285	−0.103	0.131	0.217	0.448	0.779
COVID-19 testing metric	1.132	−12.838	−0.291	0.342	2.384	13.457
Baseline traffic	1.454 × 10^−4^	−7.520 × 10^−4^	−9.066 × 10^−5^	5.327 × 10^−5^	3.842 × 10^−4^	2.282 × 10^−3^
Residential density	0.155	−0.294	0.066	0.131	0.219	0.782
% Black	0.094	−21.078	−0.129	0.653	1.951	13.017
% Native Americans	−0.174	−5.712	−1.316	−0.592	1.112	3.289
Air pollution	0.090	−0.062	0.055	0.088	0.134	0.205
% aged 65 and over	0.023	−0.043	0.012	0.025	0.035	0.067
Premature death	2.838 × 10^−5^	−6.394 × 10^−5^	−2.276 × 10^−6^	2.749 × 10^−5^	5.431 × 10^−5^	1.360 × 10^−4^
Smoking	−2.079	−12.232	−4.436	−1.957	−0.867	12.762
Diabetes	1.515	−8.721	0.693	1.487	2.210	10.722
% uninsured	0.010	−0.098	−0.001	0.013	0.027	0.055
Hospital beds	−1.011	−45.437	−11.090	0.231	6.630	57.624

## Data Availability

Publicly available datasets were analyzed in this study. This data can be found here: [https://github.com/COVID19PVI/data] (accessed on 3 June 2021).
